# 2261. Impact of Antimicrobial Stewardship Program on *Klebsiella pneumoniae* and *Escherichia coli* Antimicrobial Resistance

**DOI:** 10.1093/ofid/ofad500.1883

**Published:** 2023-11-27

**Authors:** Jose Luis Lamas Ferreiro, Judith Alvarez Otero, Ana Sanjurjo Rivo, Ignacio Enriquez de Salamanca Holzinger, Maria Fernandez Soneira, Jose Carlos de Miguel Bouzas, Irene Rodriguez Conde, Jorge Cavero, Javier de la Fuente Aguado

**Affiliations:** Ribera POVISA Hospital, Vigo, Galicia, Spain; Mayo Clinic, Rochester, Minnesota; Hospital Povisa, Vigo, Galicia, Spain; Hospital Ribera Povisa, Vigo, Galicia, Spain; Hospital Ribera Povisa, Vigo, Galicia, Spain; Hospital Povisa, Vigo, Galicia, Spain; Hospital Ribera Povisa, Vigo, Galicia, Spain; Hospital Povisa, Vigo, Galicia, Spain; Hospital Povisa, Vigo, Galicia, Spain

## Abstract

**Background:**

A decrease in antimicrobial stewardship activities has been observed in our hospital since the beginning of the pandemic. The study aim was to evaluate the impact of decrease in antimicrobial stewardship activities in *Klebsiella pneumoniae* and *Escherichia coli* acquired multidrug-resistance.

**Methods:**

The quarterly percentage of broad-spectrum antibiotic prescriptions evaluated by antimicrobial stewardship team (AMST), the quarterly consumption of broad-spectrum antimicrobials and the quarterly proportion of multidrug-resistant *K. pneumoniae* and *E. coli* were assessed between October 2016 and December 2022, with 2 periods of time, before the pandemic (October 2016-March 2020) and after the pandemic (April 2020-December 2022).Time series analysis was performed with ARIMA models to evaluate the association between decrease in AMST activities with multidrug-resistance in *K. pneumoniae* or *E. coli*. Correlation between broad-spectrum antibiotic prescriptions reviewed by AMST with broad-spectrum antimicrobials consumption and proportion of multidrug-resistance in *K. pneumoniae* or *E. coli* in urine cultures was assessed using the Pearson correlation coefficient.

**Results:**

Percentage of broad-spectrum antimicrobials evaluated by AMST was 77% in first period and 29% in second period. The mean of broad-spectrum antibiotics consumption in first period was 13.02 DDD/100 bed-days and 15.35 DDD/100 bed-days in second period. Decrease of AMST activities was associated with an 37% increase in multidrug-resistant *K. pneumoniae* in second period compared with first period (p=0.02). No differences were found in *E. coli*. Correlation between percentage of AMST-reviewed broad-spectrum antibiotics with consumption of these antibiotics was good (correlation coefficient -0,7; p< 0,01). Correlation between ASMT-reviewed broad-spectrum antibiotics and multidrug-resistant *K. pneumoniae* was moderate (correlation coefficient -0.44; p=0,02). No correlation between ASMT-reviewed broad-spectrum antibiotics and multidrug-resistant *E. coli* (correlation coefficient 0,09; p=0,6) was found.

**Conclusion:**

Decrease in antimicrobial stewardship activities was associated with high isolation of multidrug-resistant *K. pneumoniae* in urine cultures.

**Disclosures:**

**All Authors**: No reported disclosures

